# Suppression of Hypoxia-Inducible Factor 1α (HIF-1α) by Tirapazamine Is Dependent on eIF2α Phosphorylation Rather Than the mTORC1/4E-BP1 Pathway

**DOI:** 10.1371/journal.pone.0013910

**Published:** 2010-11-09

**Authors:** Jun Zhang, Ji Cao, Qinjie Weng, Rui Wu, Yan Yan, Hui Jing, Hong Zhu, Qiaojun He, Bo Yang

**Affiliations:** Institute of Pharmacology and Toxicology, Zhejiang University, Hangzhou, China; University of Cincinnati, United States of America

## Abstract

Hypoxia-inducible factor 1 (HIF-1), a heterodimeric transcription factor that mediates the adaptation of tumor cells and tissues to the hypoxic microenvironment, has attracted considerable interest as a potential therapeutic target. Tirapazamine (TPZ), a well-characterized bioreductive anticancer agent, is currently in Phase II and III clinical trials. A major aspect of the anticancer activity of TPZ is its identity as a tumor-specific topoisomerase IIα inhibitor. In the study, for the first time, we found that TPZ acts in a novel manner to inhibit HIF-1α accumulation driven by hypoxia or growth factors in human cancer cells and in HepG2 cell-derived tumors in athymic nude mice. We investigated the mechanism of TPZ on HIF-1α in HeLa human cervical cancer cells by western blot analysis, reverse transcription-PCR assay, luciferase reporter assay and small interfering RNA (siRNA) assay. Mechanistic studies demonstrated that neither HIF-1α mRNA levels nor HIF-1α protein degradation are affected by TPZ. However, TPZ was found to be involved in HIF-1α translational regulation. Further studies revealed that the inhibitory effect of TPZ on HIF-1α protein synthesis is dependent on the phosphorylation of translation initiation factor 2α (eIF2α) rather than the mTOR complex 1/eukaryotic initiation factor 4E-binding protein-1 (mTORC1/4E-BP1) pathway. Immunofluorescence analysis of tumor sections provide the *in vivo* evidences to support our hypothesis. Additionally, siRNA specifically targeting topoisomerase IIα did not reverse the ability of TPZ to inhibit HIF-1α expression, suggesting that the HIF-1α inhibitory activity of TPZ is independent of its topoisomerase IIα inhibition. In conclusion, our findings suggest that TPZ is a potent regulator of HIF-1α and provide new insight into the potential molecular mechanism whereby TPZ serves to reduce HIF-1α expression.

## Introduction

Hypoxia is a common phenomenon occurring in the majority of human tumors [1]. The microenvironment of tumors is unlike that of normal tissues because the proliferative status of the tumor cells and an irregular vascular supply result in the development of hypoxia [2], [3]. The presence of hypoxia is significantly associated with aggressive tumor progression, resistance to chemotherapy and radiation, and poor prognosis [4]. Tumor cells and tissues adapt to a hypoxic microenvironment through the activation of a number of hypoxia-related molecules and pathways, among which hypoxia-inducible factor 1 (HIF-1) is the most predominant one [5].

HIF-1 is overexpressed in common cancers and contributes to tumor growth and angiogenesis [6]. HIF-1 is a heterodimeric protein that is composed of two subunits: the O_2_-regulated HIF-1α subunit and the constitutively expressed HIF-1β subunit [7]. In normoxia, the hydroxylation of two proline residues and the acetylation of a lysine residue at its oxygen-dependent degradation domain (ODDD) promote the interaction of HIF-1α with the von Hippel-Lindau (pVHL) ubiquitin E3 ligase complex and thus marks HIF-1α for degradation by the ubiquitin-proteasome system [8]. However, under hypoxic conditions, the low availability of oxygen results in the inhibition of prolyl hydroxylase activity and, consequently, in the increase of HIF-1α stability [4]. Although the oxygen-dependent regulation of degradation is the primary mechanism of HIF-1α accumulation, HIF-1α is also known to be regulated at the translational level [4], [6]. Recent studies have shown that two distinct pathways regulate HIF-1α protein synthesis. One is the phosphorylation of eIF2α, which is responsible for a rapid inhibition of translation initiation, and the other is a reduction in the phosphorylation of 4E-BP1, a protein that is regulated by mTORC1 [9], [10].

Due to the importance of HIF-1α in cancer, targeting HIF-1α could become a novel approach in cancer therapy. It has been reported that HIF-1α-deficient cells are more susceptible to chemotherapeutic agents and radiotherapy [11]. Tirapazamine (TPZ) represents a class of hypoxia-selective cytotoxins and is currently in phase II and III clinical trials for the treatment of head and neck cancers and cervical cancer. TPZ also functions as a hypoxia-activated topoisomerase IIα poison[12]. Previous studies have shown that a number of DNA damage-inducing agents can inhibit HIF-1α protein accumulation [4], [13]. Based on these studies, we investigated whether TPZ could affect the activity of HIF-1α. Interestingly, our previous results revealed that TPZ induced a remarkable reduction in HIF-1α protein levels. In this study, we used human cervical-cancer (HeLa) cells to characterize and investigate the mechanisms involved in the reduction of HIF-1α protein levels by TPZ. The present study not only provides a better understanding of the HIF-1α signaling pathway, but also identifies the regulation of HIF-1α protein synthesis as an important target of HIF-1α-inhibitory compounds.

## Results

### TPZ inhibits the cellular accumulation of HIF-1α protein

**Figure 1:**
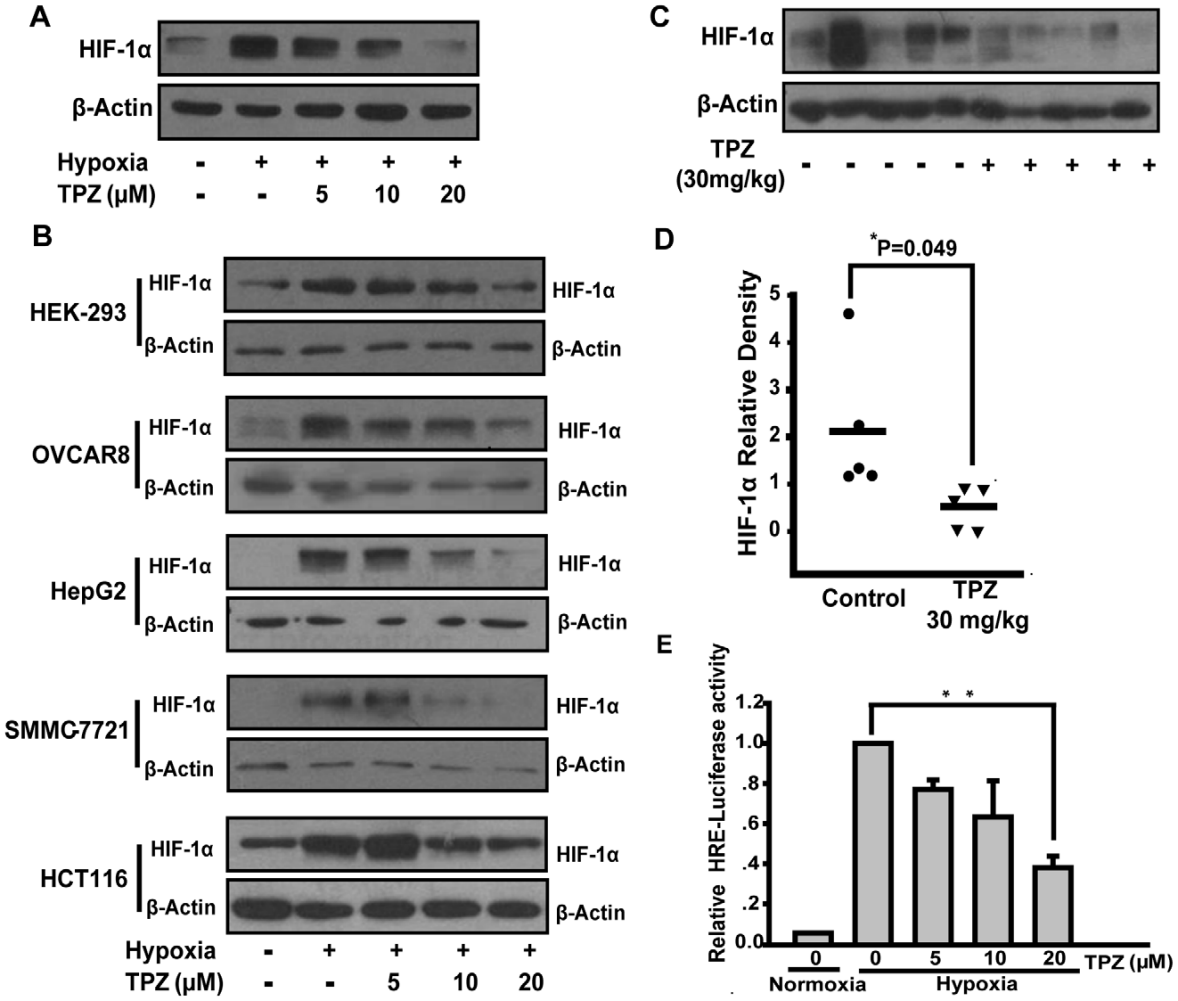
TPZ decreases hypoxia-induced HIF-1α protein accumulation. HeLa cells (A), and HEK-293, OVCAR8, HepG2, SMMC-7721 and HCT116 cells (B) were exposed to hypoxia and a gradient of concentrations of TPZ for 4 h. HIF-1α and β-actin protein levels were detected by western-blot analysis of whole-cell extracts, as described in the Materials and Methods. (C) Effect of TPZ treatment on the expression level of HIF-1α in HepG2 cell-derived tumors, as determined by immunoblot analysis. (D) Densitometry analyses of (C). (E) Hypoxia-dependent HIF-1α transcriptional activity was measured using HRE-dependent reporter assays, as described in the Materials and Methods. HeLa cells were transiently transfected with the HRE-Luc plasmid and then treated with TPZ for 8 h under hypoxic conditions. Luminescence was measured and fold stimulation was obtained by normalizing the relative luciferase activity to that of untreated cells under hypoxic conditions.

To investigate whether TPZ affects cellular HIF-1α protein expression, we used various concentrations of TPZ to treat HeLa cells under hypoxic conditions. As expected, hypoxia induced a robust accumulation of HIF-1α protein, and the addition of TPZ decreased hypoxia-induced HIF-1α protein expression in a concentration-dependent manner (Fig. 1A). Similarly, TPZ also blocked the accumulation of HIF-1α protein induced by the growth factors insulin and EGF (Fig. S1A), both of which are known to stimulate HIF-1α expression through the PI3K/Akt signaling pathway [14], [15]. Given that the inhibition of HIF-1α accumulation in hypoxic cells might be correlated with TPZ-induced cytotoxicity, parallel studies of cell viability were performed (Fig. S1B). After the HeLa cells were treated with TPZ (20 µM) for 4 h under hypoxic conditions, no significant alteration of cell viability was observed relative to the untreated control group. Next, in order to address whether the inhibition of HIF-1α by TPZ was cell line specific, we extended these studies to a diverse set of tumor cell lines with tissues of various origins, including the hepatic-cancer cell lines HepG2 and SMMC-7721, the colon-cancer cell line HCT116, the breast-cancer cell line OVCAR8 and the embryonic-kidney cell line HEK-293. Fig. 1B shows that, under hypoxic conditions, HIF-1α accumulation is suppressed by TPZ in all cell lines. Although cell-based experiments demonstrated the suppression of HIF-1α by TPZ *in vitro*, the *in vivo* HIF-1α-inhibitory activity of TPZ remained to be elucidated. Accordingly, we evaluated the effect of TPZ administration on the expression level of HIF-1α protein in HepG2 cell-derived tumors in athymic nude mice. TPZ administration was found to diminish the level of HIF-1α in the tumors (Fig. 1C and D). Consistent with the inhibition of HIF-1α accumulation, TPZ also mediated the concentration-dependent inhibition of HIF-1-mediated transcriptional activity under hypoxic conditions, as determined using a hypoxia-responsive reporter construct (Fig. 1E). The employed construct contains a *luciferase* gene under the control of the hypoxia response elements (HREs). These results collectively indicate that TPZ treatment decreases the expression of HIF-1α in *vitro* and *in vivo* and that TPZ demonstrates this activity across various human tumor cell lines and under stimulation with growth factors.

### TPZ does not affect HIF-1α mRNA expression or protein degradation, but decreases HIF-1α protein synthesis

**Figure 2:**
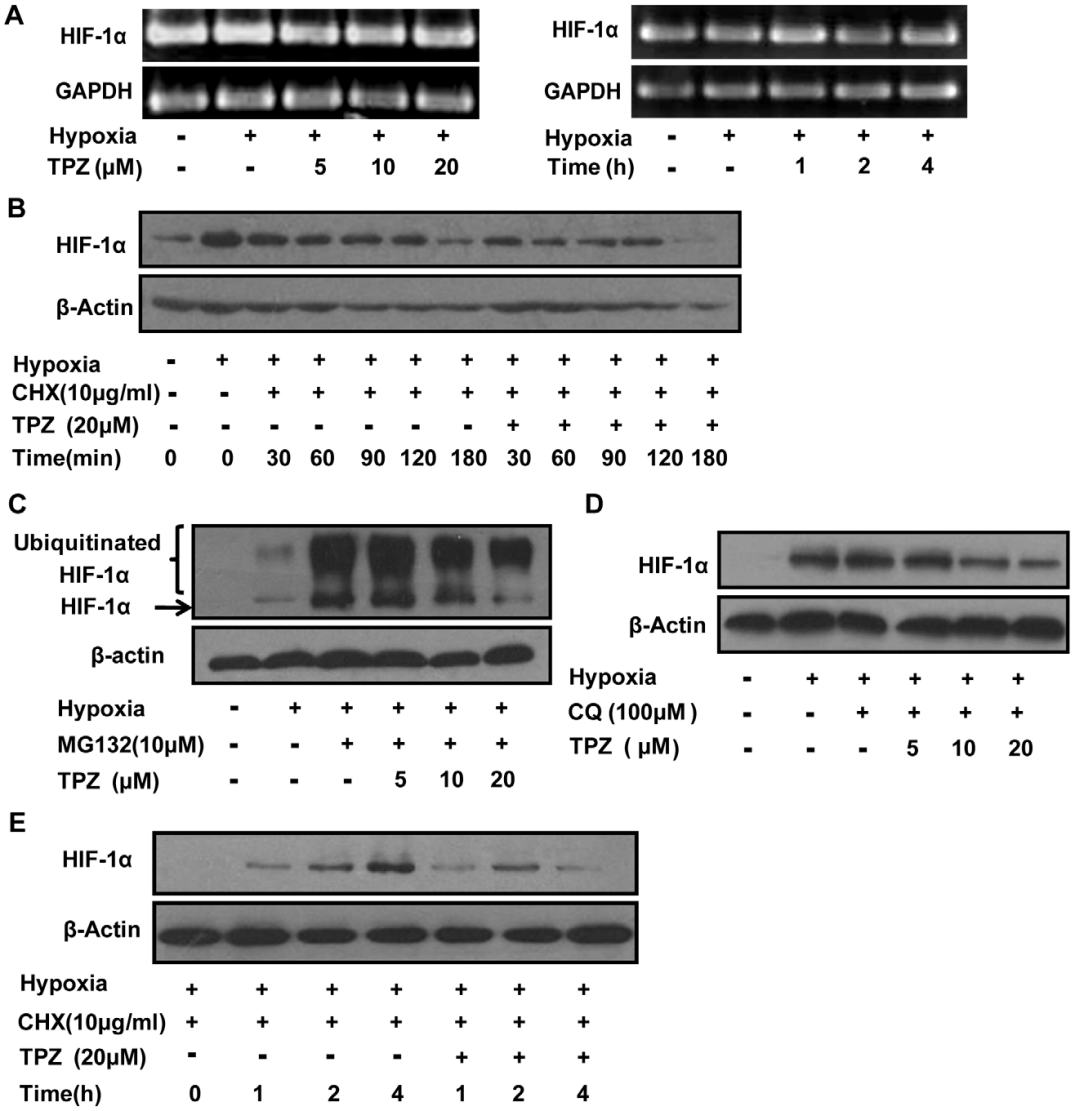
TPZ does not affect HIF-1α mRNA expression or protein degradation, but decreases HIF-1α protein synthesis. (A) HeLa cells were exposed to varying concentrations of TPZ for 4 h or a single concentration for the indicated times. Then, the total RNA was extracted and analyzed for HIF-1α mRNA expression by RT-PCR, using GAPDH as a control gene. (B) Cells exposed to hypoxia overnight were treated with cycloheximide (CHX) in the presence or absence of 20 µM TPZ for various periods, and HIF-1α protein levels were measured by western-blot analysis. HeLa cells were treated with TPZ, together with MG132 (C) or chloroquine diphosphate (CQ) (D), under the indicated conditions, followed by immunoblotting with anti-HIF-1α or anti-β-actin antibodies. Cells were pretreated for 30 min with MG132 and CQ to allow functional inhibition of the proteasome and lysosome. (E) HeLa cells were pre-incubated with CHX for 3 h in normoxic conditions and then placed in fresh medium and treated with or without 20 µM TPZ for the indicated times under hypoxic conditions. The cells were harvested and lysates were immunoblotted with an anti-HIF-1α antibody.

To ascertain whether the reduction of HIF-1α by TPZ occurs at the transcriptional level, we used reverse transcription polymerase chain reaction (RT-PCR) to study the effect of TPZ on the accumulation of HIF-1α mRNA. RT-PCR analysis indicated that HIF-1α mRNA levels were not significantly changed after TPZ treatment in HeLa cells (Fig. 2A and S2A). Furthermore, the similar results were also observed in other two cell lines (HCT116 and A549) (Fig. S2B and C). Following this observation, we investigated the effect of TPZ on HIF-1α posttranscriptional regulation. Cycloheximide (CHX) was used to prevent *de novo* protein synthesis; thus, HIF-1α levels would primarily reflect the protein degradation process. We exposed HeLa cells to CHX under hypoxic conditions in the presence or absence of TPZ at different time points and estimated the expression levels of HIF-1α. As shown in Fig. 2B, although the intensity of the HIF-1α signal was somewhat reduced in TPZ-treated cells, the degradation rates of HIF-1α were similar in treated and untreated cells. The level of HIF-1α protein in cells results from a balance between protein synthesis (translation) and its degradation [16]. To test the possibility that TPZ-mediated inhibition of HIF-1α accumulation is due to reduced HIF-1α protein synthesis, HeLa cells were pretreated with MG132 (a specific proteasome inhibitor) before the addition of TPZ and the induction of hypoxic challenge to prevent ubiquitin-dependent HIF-1α degradation. As expected, we detected a pronounced accumulation of HIF-1α protein at higher molecular weights, indicating that polyubiquitinated HIF-1α protein species form in the presence of MG132 (Fig. 2C). However, MG132 treatment did not reverse the TPZ-triggered decrease of HIF-1α protein levels (Fig. 2C). In addition to the ubiquitin-proteasome system (a non-lysosomal pathway), the lysosomal pathway is also responsible for protein degradation in cells [17], [18]. To further rule out the possibility that the lysosomal pathway participates in the inhibition of HIF-1α by TPZ, we utilized chloroquine (CQ), a lysosome inhibitor, to block nonspecific HIF-1α degradation. In keeping with the results obtained with MG132, the inhibition of protein degradation by CQ did not abolish the inhibitory effect of TPZ on HIF-1α protein levels (Fig. 2D and Fig. S2B). The findings described above indicate that TPZ interferes with the protein synthesis process and does not accelerate HIF-1α proteasome- or lysosome-mediated degradation. Together with the results of the protein degradation-rate experiments, our data also suggest that the reduction of HIF-1α protein levels by TPZ is due to the reduced synthesis of HIF-1α rather than the enhanced degradation of the protein. To more directly assess the effects of TPZ on the rate *of de novo* synthesis of the HIF-1α protein, cells were pretreated with CHX for three hours under normoxia to inhibit new protein synthesis and then incubated in fresh medium. These CHX-pretreated cells were exposed to hypoxic conditions, with or without TPZ, for different periods, and HIF-1α protein levels were analyzed by western blotting. As shown in Fig. 2E and Fig. S2C, significantly more HIF-1α protein accumulated in untreated cells than in treated cells at all times tested. This result further confirmed that TPZ decreases the rate of HIF-1α protein synthesis.

### TPZ inhibits the activation of the mTORC1/4E-BP1 pathway, which had negligible effect on the TPZ-triggered reduction of HIF-1α

**Figure 3:**
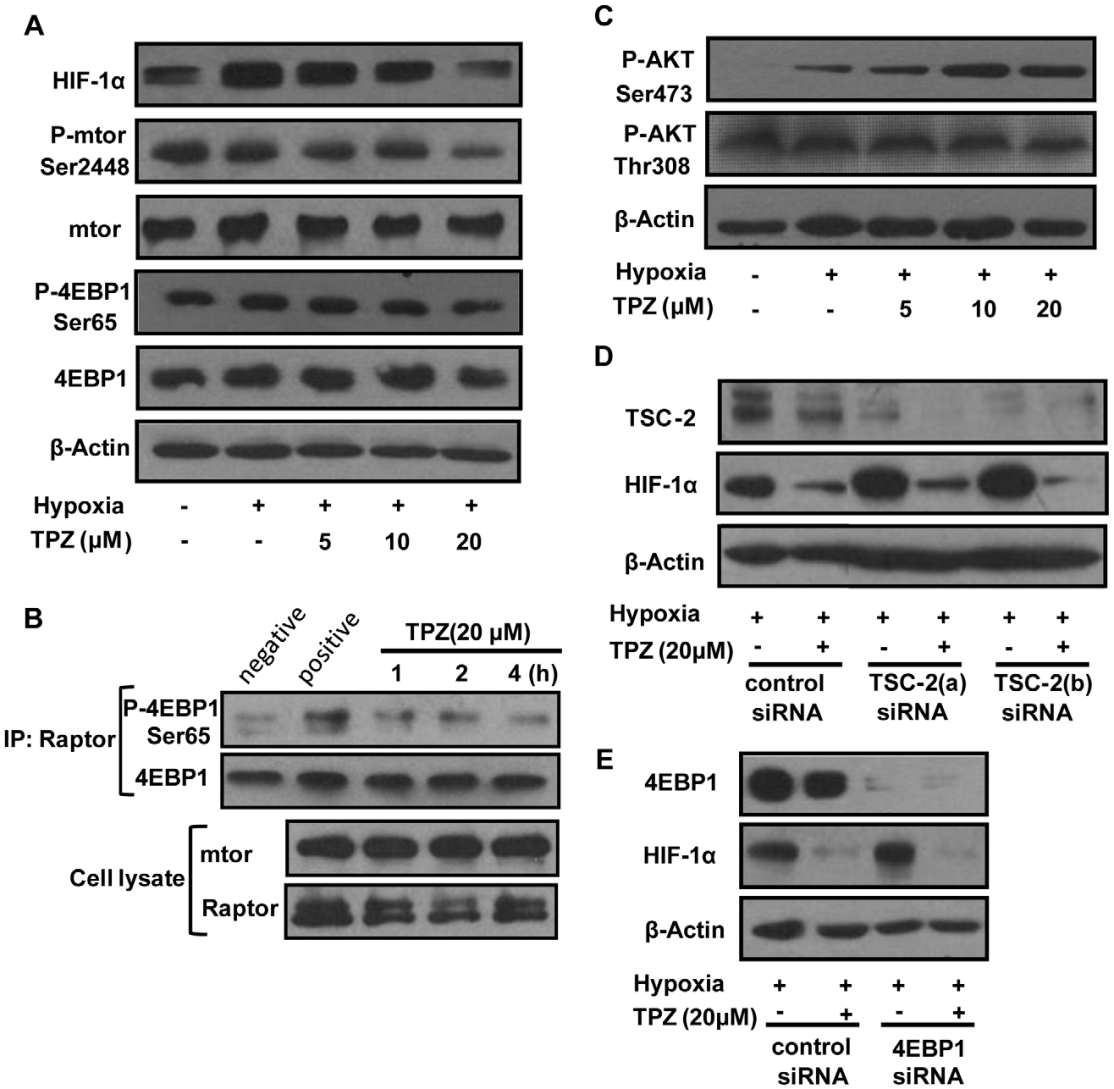
Effects of TPZ on Akt and the mTORC1 pathway in HeLa cells. (A) Cells were cultured in hypoxia for 4 h in the presence of the indicated concentrations of TPZ before western-blotting analysis. (B) Immunoprecipitates prepared from the lysates of HeLa cells with Raptor antibody were used in kinase assays with full-length 4EBP1 as the substrate. (C) Immunoblotting was used to detect the phosphorylation of Akt at Ser473 or Thr308 after TPZ treatment. HeLa cells were transfected with siRNAs specifically targeting TSC2 (D) and 4E-BP1 (E) or control siRNAs, as described in the Materials and Methods. Transfected cells were incubated with or without 20 µM TPZ for 4 h under hypoxic conditions. Proteins were detected by western-blot analysis using specific antibodies.

HIF-1α protein synthesis is regulated by mTORC1 signaling, which phosphorylates the key protein-synthesis regulator 4E-BP1; thus, the levels of phosphorylated 4E-BP1 can be used as an indicator of mTORC1 activity [19]. The phosphorylation of 4E-BP1 results in an abrogated interaction with eIF4E and thus stimulates cap-dependent translation [9]. To examine the potential involvement of this pathway in TPZ-driven events, we measured the phosphorylation status of three key proteins: mTOR, 4E-BP1 and Akt. Treatment with TPZ resulted in decreased phosphorylation of mTOR and 4E-BP1 as well as the reduction of HIF-1α accumulation (Fig. 3A), indicating an inhibition of mTORC1 signaling. The results of an *in vitro* kinase assay further confirmed that mTORC1 kinase activity was suppressed (Fig. 3B). Notably, TPZ also caused an apparent increase in Akt phosphorylation at Ser473 but induced no significant alteration in Akt phosphorylation at Thr308 (Fig. 3A). This result is in agreement with recent reports describing the activation of Akt at Ser473 as the result of mTORC1 inhibition [20], [21]. Earlier studies have shown that the TSC1-TSC2 tumor suppressor complex is a key negative regulator of mTORC1 [22]. To directly address the contribution of the mTORC1/4EBP1 pathway to the downregulation of HIF-1α by TPZ, we knocked down TSC2 and 4EBP1 expression using specific siRNAs. Although both of the siRNAs completely silenced the expression of their target genes, neither of them abolished the effect of TPZ on HIF-1α levels (Fig. 3D and 3E). Our results provide evidence that the suppression of mTORC1/4EBP1 signaling was not important for the TPZ-triggered reduction of HIF-1α protein levels. Additionally, we also found that TPZ did not affect a series of signaling pathways correlated with mTORC1 signaling, such as the Erk, AMPK, Hsp90 and Hsp70 pathways (Fig. S3).

### A direct role for eIF2α phosphorylation in HIF-1α downregulation by TPZ

**Figure 4:**
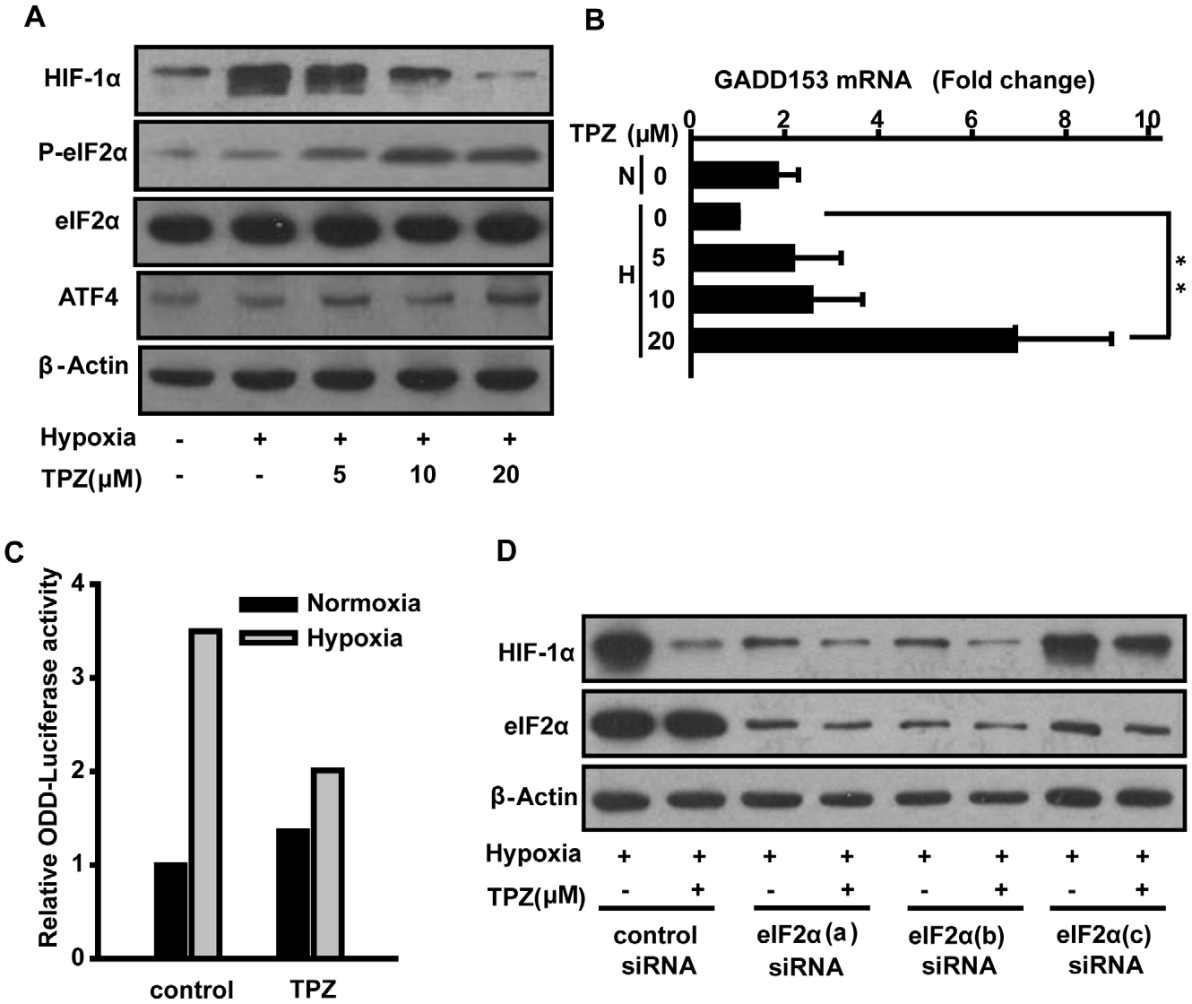
A direct role of eIF2α phosphorylation in HIF-1α downregulation by TPZ. (A) HeLa cells treated with indicated concentrations of TPZ for 4 h under hypoxic conditions. Total lysates were probed for expression of HIF-1α, p-eIF2α, and eIF2α, while β-actin served as a loading control. (B) HeLa cells were exposed to varying concentrations of TPZ for 4 h. GADD153 mRNA levels were determined by real-time PCR. The relative fold change of GADD153 mRNA compared to GAPDH mRNA in untreated cells under hypoxia was arbitrarily set as 1.0. (C) The effect of TPZ treatment to basal protein synthesis was measured using ODD-dependent reporter assays. HeLa cells were transiently transfected with the ODD-Luc plasmid and then treated with TPZ for 4 h under normoxia or hypoxia. Luminescence was measured and fold stimulation was obtained by normalizing the relative luciferase activity of cells cultured under hypoxic conditions to those of untreated cells cultured under normoxic conditions. (D) Cells were untransfected or transfected with eIF2α-targeting siRNA for two days, followed by treatment with or without 20 µM TPZ. Western blotting of cell lysates was performed using the indicated antibodies.

In addition to the mTORC1 pathway, another major pathway through which protein synthesis is controlled is the eIF2α pathway. Various cellular stress signals can induce the phosphorylation of eIF2α at Ser51 and mediate the global inhibition of protein synthesis [23], [24]. We therefore monitored the accumulation of phosphorylated eIF2α and its downstream products. TPZ induced robust eIF2α phosphorylation (Fig. 4A) and enhanced its downstream effects, including the upregulation of transcription factor 4 (ATF4) and ATF4's transcriptional target GADD153 (Fig. 4B). Furthermore, HeLa cells transiently transfected with a luciferase gene under the control of ODD were utilized to evaluate the effect of TPZ treatment on basal protein synthesis. Importantly, the ODD region of HIF-1α is sufficient for oxygen-dependent degradation [25]. As shown in Fig. 4C, TPZ exposure produced a general translational arrest of protein synthesis. In subsequent experiments, we tested whether the phosphorylation of eIF2α is required for the TPZ-dependent inhibition of HIF-1α protein synthesis by transfecting cells with siRNA targeting eIF2α. The knockdown of eIF2α attenuated basal HIF-1α protein levels and partially reversed the effects of TPZ on HIF-1α translation repression (Fig. 4D). Thus, TPZ induced down-regulation of HIF-1α is largely eIF2α phosphorylation dependent.

### TPZ reduces HIF-1α protein levels only under hypoxic conditions, and this activity is independent of TPZ's inhibition of topoisomerase II

**Figure 5:**
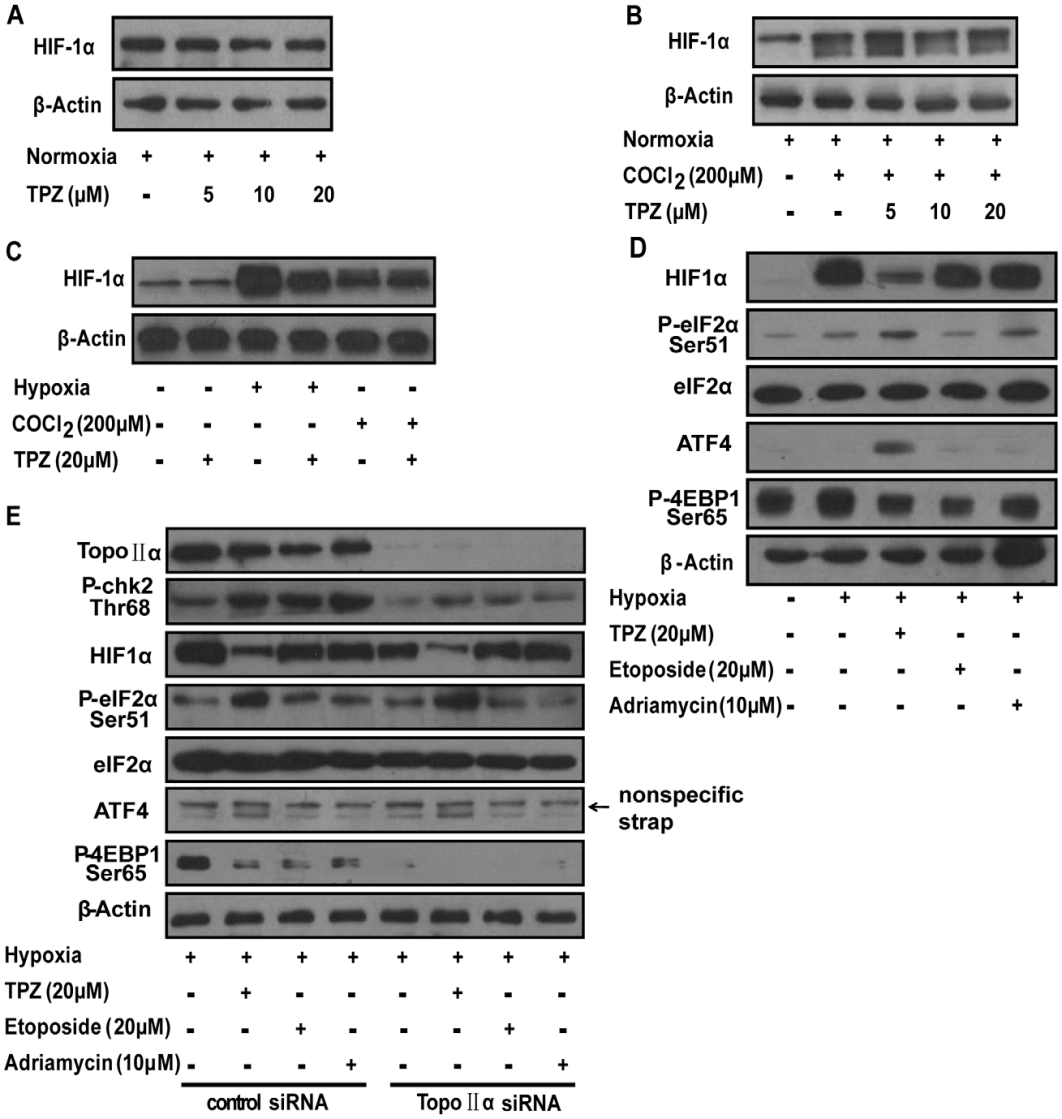
TPZ reduces HIF-1α protein only under hypoxic conditions, and its activity is independent of its topoisomerase II inhibition. (A) HeLa cells were exposed to normoxic conditions for 4 h in the presence of TPZ. (B) Cells were treated with varying concentrations of TPZ for 4 h, as indicated, in the presence of the hypoxia-mimetic agent cobalt chloride (CoCl_2_). (C) HeLa cells were treated for 4 h in normoxia, hypoxia or with CoCl_2_ in the presence of TPZ at the indicated concentrations. (D) Cells were treated with 20 µM TPZ, 20 µM etoposide and 10 µM adriamycin for 4 h. (E) Topoisomerase IIα was knocked down using siRNA, as described in the Materials and Methods, followed by drug treatment (20 µM TPZ, 20 µM etoposide and 10 µM adriamycin). Proteins were detected by western-blot analysis.

TPZ, a bioreductive drug, is selectively toxic to hypoxic cells [26]. In order to examine whether the effect of TPZ on the expression of HIF-1α is dependent on oxygen concentrations, we tested the effect of TPZ on HIF-1α expression under aerobic conditions. The addition of TPZ to the culture medium failed to abrogate the expression of HIF-1α in aerobic HeLa cells (Fig. 5A). HIF-1α accumulation induced by the prolyl hydroxylase inhibitor cobalt chloride (CoCl_2_), a well-characterized hypoxia mimetic agent [3], was unaffected by normoxia and treatment with TPZ (Fig. 5B). All of these findings collectively indicate that TPZ decreases the cellular accumulation of HIF-1α protein only under hypoxic conditions (Fig. 5C).

Additionally, TPZ has been demonstrated to be a hypoxia-activated topoisomerase II poison [12]. Here, in an attempt to explore whether topoisomerase II inhibition is required for the reduction of HIF-1α protein levels by TPZ, we employed two potent topoisomerase II inhibitors: etoposide and adriamycin [27]. As shown in Fig. 5D, only TPZ treatment resulted in the downregulation of HIF-1α protein levels, while etoposide and adriamycin had no effect on the expression of HIF-1α. Thus, there is no direct correlation between topoisomerase II inhibition and HIF-1α reduction. Furthermore, we tested the HIF-1α protein levels, with or without treatment with TPZ, in HeLa cells that were either untransfected or transfected with siRNA targeting topoisomerase IIα (Fig. 5E). Topoisomerase IIα-targeting siRNA completely silenced the expression of topoisomerase IIα and disrupted phosphorylated Chk2 (a critical mediator of topoisomerase II-induced DNA damage) [28], indicating that topoisomerase IIα was inactive in these cells. The transfection of topoisomerase IIα-targeting siRNA into HeLa cells did not exert any detectable effects on the TPZ-mediated reduction in HIF-1α protein accumulation (compare lane 2 with lane 6). These findings suggest that the TPZ-induced reduction in HIF-1α expression is a topoisomerase II-independent phenomenon.

### *In vivo* efficacy of TPZ in an athymic nude mouse model

**Figure 6:**
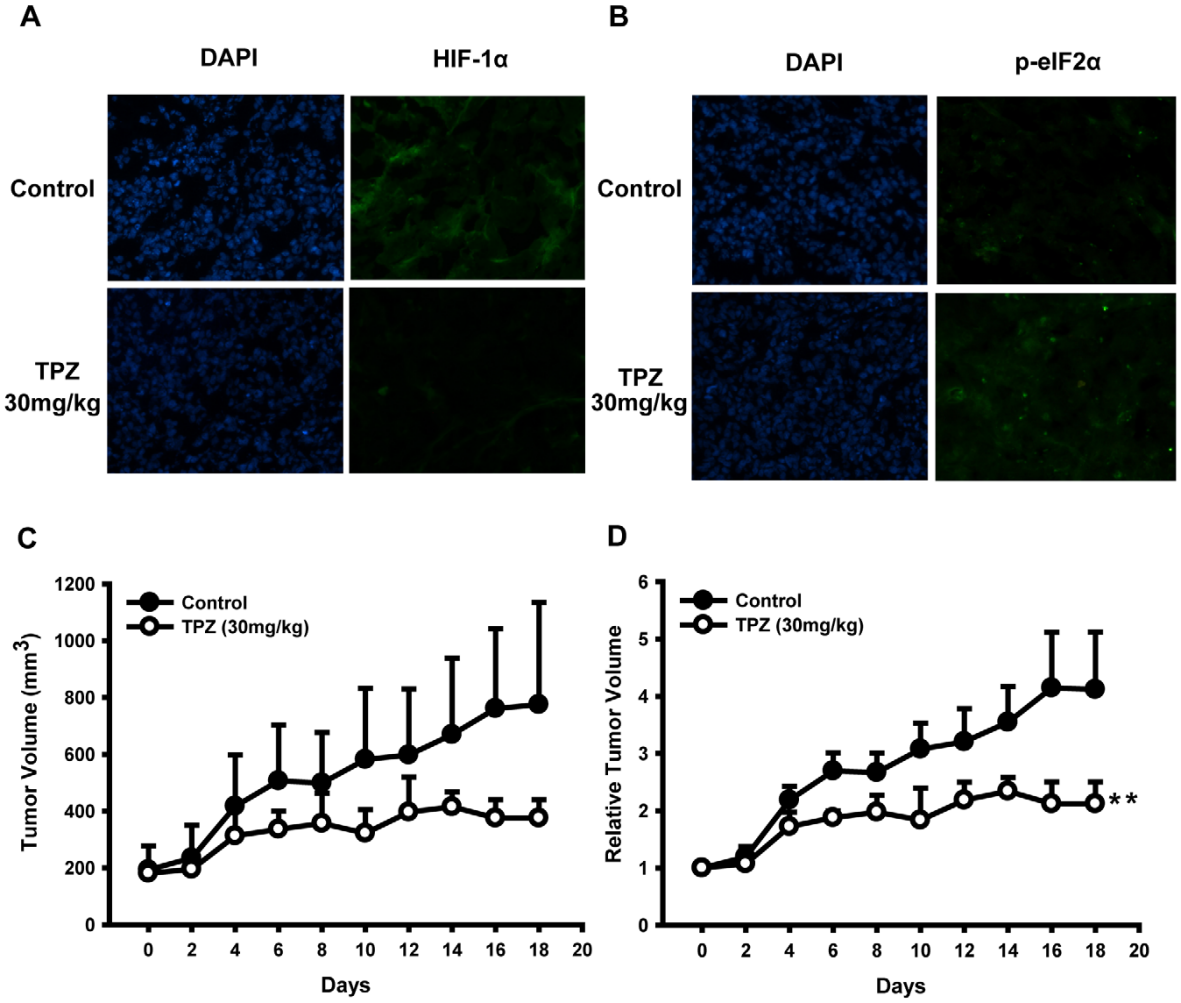
The effect of TPZ on HepG2 human xenograft models. The mice transplanted with HepG2 human xenograft were randomly divided into two groups and given injection of TPZ (30 mg/kg) or vehicle every 2 days. Representative photomicrographs (magnification, x100) showing immunofluorescence staining for HIF-1α (A) and p-eIF2α (B) in tumor sections of vehicle–treated and TPZ-treated mice. The immunostaining data were confirmed in two or more specimens of each group. (C) Tumor volume are expressed as mean ± SD. (D) Relative tumor volume are expressed as mean ± SD. (**p<0.01, relative to vehicle group).

Because our previous results revealed that TPZ modulate HIF-1α protein synthesis by phosphorylation of eIF2α *in vitro*, we next determined whether these results could be translated into an *in vivo* xenograft model. A short times (9 days) TPZ administration was observed to decrease the expression level of HIF-1α protein (Fig. 1C and D). This study was confirmed by performing immunofluorescence of HIF-1α and p-eIF2α proteins in tumor sections of both groups of animals. TPZ caused a reduction in HIF-1α-positive staining but induced an apparent increase in p-eIF2α-positive staining in tumor tissues of animals (Fig. 6A and B), which were consistent with our *in vitro* studies.

Considering several articles have been reported that HIF-1α plays an active role in tumor progression and transplantation of tumors lacking HIF into immunodeficient mice results in decreased tumor growth [11], [29], we next asked whether the observed effect of TPZ has any impact on tumor physiology. As expected, the administration of TPZ (30 mg/kg every 2 days) inhibited the tumor growth, with T/C value 51.5% and inhibition rate 36.5%. The relative tumor volume (RTV) of TPZ-treated group was remarkably decreased from that of vehicle group (p<0.007) (Fig. 6D). Collectively, these data further suggest that TPZ has the potential to inhibit the tumorigenicity of HepG2 cells *in vivo*.

## Discussion

HIF-1 is constitutively upregulated in various types of cancer and plays a major role in tumor progression [11]. In addition to serving as a surrogate marker of tumor responsiveness to therapy, HIF-1 has rapidly attracted interest both for its potential role as a therapeutic target and its involvement in fundamental physiological and pathophysiological processes, including angiogenesis, resistance to chemotherapy and radiotherapy, tumor invasiveness and poor prognosis of cancer patients [5], [29], [30]. Due to the potential role of HIF-1 as a target for cancer therapy, the development of small-molecule HIF-1 inhibitors represents a major challenge in the field of cancer treatment. This study is the first to demonstrate that TPZ, a hypoxia-selective cytotoxin, induces an unexpected downregulation of hypoxia-dependent and mitogen-dependent HIF-1α accumulation in human tumor cell lines originating from various tissues. Moreover, we assessed the toxicity of TPZ within a relatively short treatment time (4 h), thus, the observed inhibition of HIF-1α accumulation by TPZ is not attributable to cell death.

The accumulation of HIF-1α is regulated through both protein degradation and protein synthesis. In the present study, we found that TPZ inhibits HIF-1α accumulation without affecting HIF-1α protein degradation and mRNA levels. These observations support the hypothesis that the TPZ-dependent reduction of HIF-1α accumulation is due to the decrease of de novo HIF-1α protein synthesis.

HIF-1α protein translation has emerged as an important regulatory mechanism of HIF-1α-inhibitory compounds. HIF-1α protein translation is known to be regulated through the mTORC1 [22]pathway. The mTORC1 pathway regulates translation with such downstream effectors as 4E-BP1 and ribosomal protein S6 kinase (p70S6K) [22]. Hypophosphorylated 4E-BP binds eIF4E, thereby preventing its association with eIF4G and inhibiting cap-dependent translation [9]. In the present study, TPZ was found to inhibit the phosphorylation of mTOR and 4E-BP1, which accompanied the loss of HIF-1α expression. Therefore, given the key role of this pathway in the regulation of HIF-1α translation, our results strongly suggest that TPZ-induced suppression of the mTORC1 pathway might serve to inhibit HIF-1α protein translation. However, the near-complete abrogation of TSC2 and 4E-BP1 expression through siRNA-mediated knockdown did not abolish the effect of TPZ on HIF-1α levels. These results indicate that the suppression of mTORC1/4E-BP1 signaling had a negligible effect on the TPZ-triggered reduction of HIF-1α. Currently, a number of compounds with different anticancer molecular targets, such as YC-1 [29] and 2-methoxyestradiol [31], have been reported to reduce hypoxia-induced HIF-1α accumulation through a translation-dependent mechanism. Most of these compounds were demonstrated to suppress the mTORC1/4E-BP1 pathway; however, neither siRNA nor the transfection of dominant-negative mutants was used to further confirm that the reduction of HIF-1α was due to interference with the mTORC1/4E-BP1 pathway. Notably, Garcia-Maceira et al. [6] reported that silibinin was more effective in inhibiting HIF-1α accumulation than rapamycin, which suggests the participation of an additional mechanism in addition to repression of the mTORC1/4E-BP1 pathway. Accordingly, it is reasonable to believe that the reduction of HIF-1α is not linked to the repression of mTORC1 signaling. Therefore, further studies are needed to explore additional mechanisms that are related to protein synthesis in addition to mTORC1/4E-BP1 inactivation.

A recent study reported that the phosphorylation of eIF2α might play a role in HIF-1α translational regulation. Normally, during protein synthesis, the exchange of eIF2α-GDP to eIF2α-GTP is required for the re-formation of ternary translation initiation complexes [32], [33]. For eIF2α-GDP to be recycled, eIF2B is required. Phosphorylated eIF2α at serine 51 binds to eIF2B with high affinity. Thus, eIF2B cannot recycle eIF2α-GDP, thereby leading to global protein translation arrest. Decreased initiation, paradoxically, leads to the increased expression of ATF4 [34]. We have shown that treatment with TPZ upregulates the phosphorylation of eIF2α and its downstream effector ATF4, which parallels the reduction of HIF-1α protein accumulation. ATF4 induces the expression of numerous genes, such as GADD153 and VEGF [33]. VEGF is considered a classic HIF-1 target gene. Measuring the mRNA expression of VEGF by PCR, we found that it is not affected by TPZ treatment, while the mRNA level of PHD3 (a HIF-1 target gene) is reduced and the GADD153 mRNA level is enhanced (Fig. 4B and S4). PCR results further suggested the potential connection between TPZ-triggered eIF2α phosphorylation and HIF-1α inhibition. Indeed, the knockdown of eIF2α attenuated basal HIF-1α protein levels and partially reversed the effects of TPZ on HIF-1α translational repression. Furthermore, immunofluorescence analysis revealed that the down-regulation of HIF-1α was observed to be concomitant with increased eIF2α phosphorylation in TPZ-treated cell-originated tumors. In conclusion, these data indicate that TPZ induces the phosphorylation of eIF2α and that this effect likely accounts for its HIF-1α inhibitory activity.

Of note, stress to the endoplasmic reticulum (ER) activates a set of signaling pathways collectively termed the unfolded protein response (UPR) [24], [34]. One branch of the UPR is initiated by activation of the ER-stress sensor PERK, an eIF2α kinase [35]. Therefore, we sought to examine whether TPZ was able to cause ER stress. Unexpectedly, unlike tunicamycin (Tm, an agent that can induce ER stress), TPZ did not upregulate the canonical UPR indicator Grp78 and induce the appearance of spliced Xbp1 mRNA (Fig. S5), indicating that TPZ selectively engages in the translational-control branch of the UPR by inducing eIF2α phosphorylation without causing ER stress or activating the transcription-dependent branch of the UPR.

Certain topoisomerase II inhibitors have been found to inhibit HIF-1 activity, such as NSC644221 [16] and adriamycin [13], [36]. Particularly, NSC644221is known to inhibit HIF-1α protein expression in a cell type-specific and topoisomerase II-dependent manner. This potential correlation between HIF-1α and topoisomerase II attracted our interest. We observed that etoposide and adriamycin had no effect on the expression of HIF-1α, which is consistent with recent studies [13], [36]. Additionally, the silencing of topoisomerase IIα did not abolish the TPZ-mediated reduction of HIF-1α protein accumulation. Unexpectedly, our studies revealed a lack of direct correlation between topoisomerase II inhibition and HIF-1α reduction, and TPZ-driven HIF-1α reduction was not a consequence of TPZ-mediated topoisomerase II inhibition, suggesting that TPZ targets both HIF-1α and topoisomerase II. Moreover, in topoisomerase IIα knockdown cells, TPZ was able to decrease HIF-1α expression, which was consistent with the change in phosphorylation of eIF2α and with mTORC1/4E-BP1 inhibition. However, etoposide and adriamycin treatments, concurrently performed, had no effect on the expression of HIF-1α regardless of mTORC1/4E-BP1 inhibition (Fig. 5E). Similar results were observed in cells untransfected with siRNA (Fig. 5D). These findings further suggest that the inhibitory effect of TPZ on HIF-1α protein is dependent on the phosphorylation of eIF2α rather than the mTORC1/4E-BP1 pathway.

Transplantation of tumors lacking HIF into athymic mice resulted in increased responsiveness to the treatment with carboplatin, etoposide and ionizing radiation, all of which induce DNA damage, primarily by double-strand breaks. Recent findings provided evidence that double-strand break repair enzymes (potential targets of HIF-1) are associated with responsiveness to tumor therapy [11]. In this study, we found that TPZ acts in a novel manner to inhibit HIF-1 activity by stimulating the phosphorylation of eIF2α but not mTORC1/4E-BP1 repression. The new mode of action exhibited by TPZ may, in part, explain why the combination of TPZ with conventional anticancer treatments (IR, VP-16, cisplatin, etc.) is particularly effective [37], [38], [39]. The present study not only provides a new understanding of the HIF-1α-inhibitory activity and the underlying mechanisms of TPZ, but also underscores its potential for further research and development as an HIF-1α inhibitor, alone or in combination with other agents, to produce even stronger anticancer activities.

## Materials and Methods

### Ethics Statement

This study was carried out in accordance with the National Institute of Health Guide for the Care and Use of Laboratory Animals. The protocol was approved by the Committee on the Ethics of Animal Experiments of the Zhejiang University (Permit Number: Zju2009101003 and Zju2010101032).

### Reagents and antibodies

TPZ was supplied by Topharman Shanghai Co., Ltd. The compound was dissolved in dimethylsulfoxide (DMSO) (40.0 mM stock solution) and stored at −20°C. The stock solution was freshly diluted with medium before use. The final DMSO concentration did not exceed 0.1% (v/v). MG132, cycloheximide (CHX), cobalt chloride (CoCl_2_), chloroquine diphosphate salt (CQ), etoposide and adriamycin were obtained from Sigma-Aldrich (St. Louis, MO, USA). Epidermal growth factor (EGF) was purchased from Invitrogen (Carlsbad, CA, USA). Insulin was purchased from Sigma–Aldrich. The primary antibody for HIF-1α was purchased from the BD Transduction Laboratories (San Jose, CA, USA). The primary antibodies for p-Akt (Ser473), p-Akt (Thr308), Akt, p-4E-BP1 (Ser65), eIF2α, p-eIF2α (Ser51), CREB-2 (ATF4), topoisomerase IIα, TSC2, Raptor, β-actin, Hsp90, Hsp70 and p-Erk were from Santa Cruz Biotechnology (Santa Cruz, CA, USA). The primary antibody for 4E-BP1, LC3B, p-AMPK(Thr172), mTOR and p-mTOR (Ser2448) were obtained from Cell Signaling Technology (Beverly, MA, USA). The primary antibody for p-chk2 (T68) was from R&D Technology. Secondary antibodies for rabbit IgG, goat IgG and mouse IgG were from Santa Cruz Biotechnology. Enhanced chemiluminescence, a western blot detection reagent, was obtained from Pierce Chemical (Rockford, IL, USA).

### Cell culture

Human hepatic-cancer HepG2 and SMMC-7721 cells, cervical-cancer HeLa cells, colon-cancer HCT116 cells, breast-cancer OVCAR8 cells and embryonic-kidney HEK-293 cells were obtained from the Cell Bank of the China Science Academy (Shanghai, China). Cells were normally cultured with the Cell Bank-required medium at 37°C in a humidified atmosphere with 5% CO_2_. All media were supplemented with heat-inactivated fetal bovine serum (FBS) (Gibco BRL, Grand Island, NY) plus penicillin (100 units/ml) and streptomycin (100 µg/ml). Moderately hypoxic conditions (0.6% O_2_) were achieved by putting cells in a hypoxia incubator (Forma Scientific, Inc., Marietta, OH) filled with a mixture of 0.6% O_2_, 5% CO_2_ and 94.4% N_2_.

### Western blotting

Exponentially growing cells (70–80% confluence) in complete medium were treated with different concentrations of TPZ and/or other agents for the indicated times under normoxic or hypoxic conditions. The cells were collected and lysed in 2X SDS gel-loading buffer [24 mM Tris-HCl (pH 6.8), 0.02% mercaptoethanol, 4% SDS, 0.4% bromphenol blue, 20% glycerol] and then boiled for 10–15 minutes. Equal volumes of cell lysates were resolved on 8%–15% SDS-PAGE gels, and the proteins were transferred to PVDF membranes (Pierce Chemical). The blots were incubated with the indicated primary antibodies and then the appropriate horseradish peroxidase-conjugated secondary antibodies. The signals were visualized by the ECL Plus western-blotting detection system (Pierce Chemical).

### Reverse transcription-PCR

Total RNA from HeLa cells was isolated using the Trizol reagent (Sangon Biotech Co., Ltd), and cDNA was synthesized using 2 µg of total RNA with random hexamer primers and the Moloney murine leukemia virus reverse transcriptase (M-MuLV RT) (Fermentas International Inc., Burlington, Ontario, Canada). The conditions used for reverse transcription-PCR were as follows: 10 min at 25°C, 60 min at 42°C and 15 min at 72°C. The cDNA was subjected to PCR amplification using the following forward and reverse primer sets: HIF-1α, forward primer: 5′-TCACCACAGGACAG TACAGGATGC-3′ and reverse primer: 5′-CCAGCAAAGTTAAAGCATCAGG TTCC-3′; VEGF, forward primer: 5′-AGGAGGGCAGAATCATCACG-3′ and reverse primer: 5′-CAAGGCCCACAGGGATTTTCT-3′; Xbp1, forward primer: 5′-CCTTG TAGTTGAGAACCAGG-3′ and reverse primer: 5′-GGGGCTTGGTATATATGTG G-3′; GRP78, forward primer: 5′-GTATTGAAA CTGTAGGAGGTGTC-3′ and reverse primer: 5′-TATTACAGCACTAGCAGATCAG-3′; GADD153, forward primer: 5′- GCACCTCCCAGAGCCCTCACTCTCC-3′ and reverse primer: 5′-GTCTACTC CAAGCCTTCCCCCTGCG-3′; PHD3, forward primer: 5′-TCAAC TTCCTCCTGTC CCTCATC-3′ and reverse primer: 5′-GCGAACATAACCTGTCCCATTTC-3′; GAPDH, forward primer: 5′-GTCATCCATGACAACTTTGG-3′ and reverse primer: 5′-GAGCTTGA CAAAGTGGTCGT-3′. The housekeeping gene GAPDH was used as the internal standard. PCR products were separated on 1.0% agarose gel and visualized by ethidium bromide staining. Gels were photographed using a Gel DOC 2000 image analyzer (Bio-Rad, Hercules, CA, USA). The quantitative real-time RT-PCR analysis was performed by TAKARA SYBR Premix EXTaqTM. The reaction mixtures containing SYBR Green were composed following the manufacturer's protocol. The cycling program was 95°C for 30 s, 58°C or 70°C (GADD153) for 20 s, and 72°C for 30 s followed by 40 cycles using an Eppendorf epGradient Mastercycler (Eppendorf, Hamburg, Germany).

### Small interfering RNA (siRNA) transfection

siRNA duplexes against human 4E-BP1, TSC2, topoisomerase IIα, eIF2α and control scrambled siRNA were synthesized by Shanghai GenePharma Co., Ltd. The sense strands of siRNAs against 4E-BP1, TSC2, topoisomerase IIα, and eIF2α were as follows: 4E-BP1: GGUACCAGG AUCAUCUAUGTT [40]; TSC2: CAAUGAGUCACAGUCCUUU GA (a) and AAAGU UCACCUACUGCUGGCA (b) [41]; topoisomerase IIα: GGUAUUCCUGUUGUUGAAC [42]; eIF2α: GGCUUGUUAUGGUUAUGAA(a), CCUCGGUAUGUAAUGACUA(b) and GAGAGGCUUGAAAGAGAAA(c) [43]. Briefly, HeLa cells were seeded into six-well plates and grown to 30–40% confluence before transfection. Cells were transfected with double-stranded siRNAs (at final concentrations of 80–100 nM) for 4–6 h by the oligofectamine method, according to the manufacturer's protocol (Invitrogen), and incubated in fresh media containing 10% FBS for the indicated time before starting an experiment.

### Luciferase reporter assays

HeLa cells were seeded into 96-well plates and grown to 80% confluence before transfection. Cells were cotransfected with renilla luciferase (internal control for transfection efficiency) and plasmids HRE-luciferase-pGL3 or ODD-luciferase-pcDNA3 (Addgene, Inc.) encoding a firefly luciferase reporter driven by a promoter containing an HRE or ODD, respectively, using Lipofectamine 2000 reagent (Invitrogen) according to the manufacturer's instructions. Luciferase activity was measured using the Dual-Luciferase reporter assay system (Promega, Madison, Wis., USA). In the assay, firefly luciferase activity was normalized by renilla luciferase.

### Transient transfection assays

HeLa cells were seeded into 6-well plates in standard growth medium. After an overnight culture, the cells were transiently transfected with 4 µg Xbp1-DBD-*venus* plasmid (supplied by Professor Jia Li), DNA using Lipofectamine 2000 (Invitrogen), according to the manufacturer's instructions. Xbp1-DBD-*venus* (the gene encoding venus - a variant of the green fluorescent protein) acts as an indicator of ER stress, as described [44]. Under ER stress, the transcripts from Xbp1-DBD-*venus* constructs were spliced. The spliced mRNA was translated into an Xbp1-*venus* fusion protein, which can be detected by its fluorescence.

### Immunoprecipitation and mTORC1 kinase assays

Immunoprecipitates were prepared as previously described [45]. For immunoprecipitation experiments, the lysis buffer contained 0.3% CHAPS instead of 1% Triton in order to preserve the integrity of the mTOR complexes. First, 10 µL of Raptor antibody was added to the cleared cellular lysates and incubated for 90 min. 25 µl of Protein A/G PLUS-Agarose (Santa Cruz Biotechnology) was then added and incubated for 1 h. Immunoprecipitates captured with Protein A/G PLUS-Agarose were washed four times with the CHAPS Lysis Buffer and once with the mTORC1 kinase buffer (25 mM Hepes pH 7.5, 100 mM potassium acetate, 1 mM MgCl_2_). For kinase reactions, immunoprecipitates were incubated in a final volume of 40 µl for 20 min at 37°C in the mTORC1 kinase buffer containing 500 ng 4EBP1 fusion protein (Santa Cruz Biotechnology) and 500 µM ATP. After centrifugation at 6000 rpm for 1 min, the supernatant was removed from the Protein A/G PLUS-Agarose and analyzed by immunoblotting [45].

### Determination of HIF-1α expression *in vivo*

Five- to six- week-old BALB/c female athymic mice (weight, 18–22 g) were supplied by the Shanghai Laboratory Animal Center, Chinese Academy of Sciences. Tumors were established by injection of HepG2 cells (5×10^6^ cells per animal, subcutaneously injected into the armpit of the athymic mice) into mice. Mice were intraperitoneally administrated TPZ dissolved in physiological saline (30 mg/kg) once every two days for 10 days. At the termination of experiment, animals were sacrificed and tumor tissues were harvested. From the harvested tissues, lysates were prepared and frozen tumor sections were prepared on slides. The lysates were used to evaluate the expression levels of HIF1α by western blotting and frozen tumor sections were immediately processed for immunofluorescence analysis.

### Measurement of *In Vivo* Activity

Human hepatic cancer HepG2 xenografts were inoculated in nude mice as described above. The mice were randomized to control and treated groups, and received vehicle (physiological saline) and TPZ (30 mg/kg, i.p. administration) every 2 days for indicated days. The size of tumors were measured individually every two days with microcalipers. Tumor volume (V) was calculated as V  =  (length×width) 2/2. The individual relative tumor volume (RTV) was calculated as follows: RTV  =  V_t_/V_0_, where V_t_ is the volume on each day of measurement and V_0_ is the volume on the day of initial treatment. Therapeutic effect of compound was expressed in terms of T/C% and the calculation formula is T/C (%)  =  mean RTV of the treated group/mean RTV of the control group ×100%.

### Immunofluorescence

Immunofluorescence was performed as described previously [46]. Frozen sections (10 µm thick) of HepG2 tumor were incubated with anti-HIF-1α (1∶100) or anti-p-eIF2α (1∶50) at 4° overnight followed by 60 min incubation with a secondary antibody at room temperature. Nuclei were visualized by staining DAPI. Fluorescence images were acquired with fluorescence microscope.

### Statistical analysis

Data were presented as means ± SD, and the significance of the differences between the values of the groups was determined with an unpaired Student's *t-*test. Differences were considered significant at P<0.05.

## Supporting Information

Figure S1 (A) HeLa cells were exposed to 20 µM TPZ after being stimulated by epidermal EGF (100 ng/mL) or insulin (80 U/L) for 4 h at hypoxia. Whole-cell extracts were subjected to immunoblot analysis. (B) Cells were treated with TPZ under hypoxic conditions for 4 h and viewed by microscope. Cell viability was not significantly altered.
(0.90 MB TIF)

Figure S2 (A) HeLa cells were exposed to varying concentrations of TPZ for 4 h or a single concentration for the indicated times. HIF-1α mRNA levels were determined by real-time PCR. The relative fold change of HIF-1α mRNA compared to GAPDH mRNA in untreated cells under normoxia was arbitrarily set as 1.0. HCT116 cells (B) and A549 cells (C) were exposed to indicated concentrations of TPZ for 4 h at hypoxia. HIF-1α mRNA levels were determined by real-time PCR. The relative fold change of HIF-1α mRNA compared to GAPDH mRNA in untreated cells was arbitrarily set as 1.0. (D) HeLa cells were treated with TPZ, together with chloroquine diphosphate (CQ), under the indicated conditions. The cells were harvested and lysates were immunoprecipitated with an LC3B antibody. The conversion of LC3-I to the lower migrating form LC3-II have been used as a indicator of functional inhibition of the lysosome. (E) HepG2 cells were pre-incubated with CHX for 3 h in normal conditions and then placed in fresh medium and treated with or without 20 µM TPZ for the indicated times under hypoxic conditions. The cells were harvested and lysates were immunoblotted with an HIF-1α antibody.
(0.91 MB TIF)

Figure S3 TPZ does not affect the Erk and AMPK pathways and Hsp-family proteins. (A–C) HeLa cells were treated with the indicated concentrations of TPZ at hypoxia for 4 h. Then, the cells were collected and detected for western blotting using specific antibodies.
(0.43 MB TIF)

Figure S4 Effects of TPZ on HIF-1α target genes. (A–B) HeLa cells were exposed to varying concentrations of TPZ for 4 h. PHD3 and VEGF mRNA levels were determined by real-time PCR. The relative fold changes of PHD3 and VEGF mRNA compared to GAPDH mRNA in untreated cells under hypoxia was arbitrarily set as 1.0.
(0.24 MB TIF)

Figure S5 TPZ does not cause ER stress or activate the transcription-dependent branch of the UPR. (A–B) RT-PCR analysis showing induction of UPR targets Grp78 and the appearance of spliced Xbp1 by Tm, but not by TPZ treatment of HeLa cells. (C) HeLa cells were transfected with Xbp1-DBD plasmid and then treated with 20 µM TPZ or 10 µg/mL Tm for 4 h under hypoxic conditions. Fluorescent images were obtained by fluorescence microscope.
(2.24 MB TIF)
