# Supplementary figures and images for: Suppression of Hypoxia-Inducible Factor 1α (HIF-1α) by Tirapazamine Is Dependent on eIF2α Phosphorylation Rather Than the mTORC1/4E-BP1 Pathway
Source: PLoS One. 2010 Nov 9;5(11):e13910. doi: 10.1371/journal.pone.0013910 (PMC2976688; doi:10.1371/journal.pone.0013910)

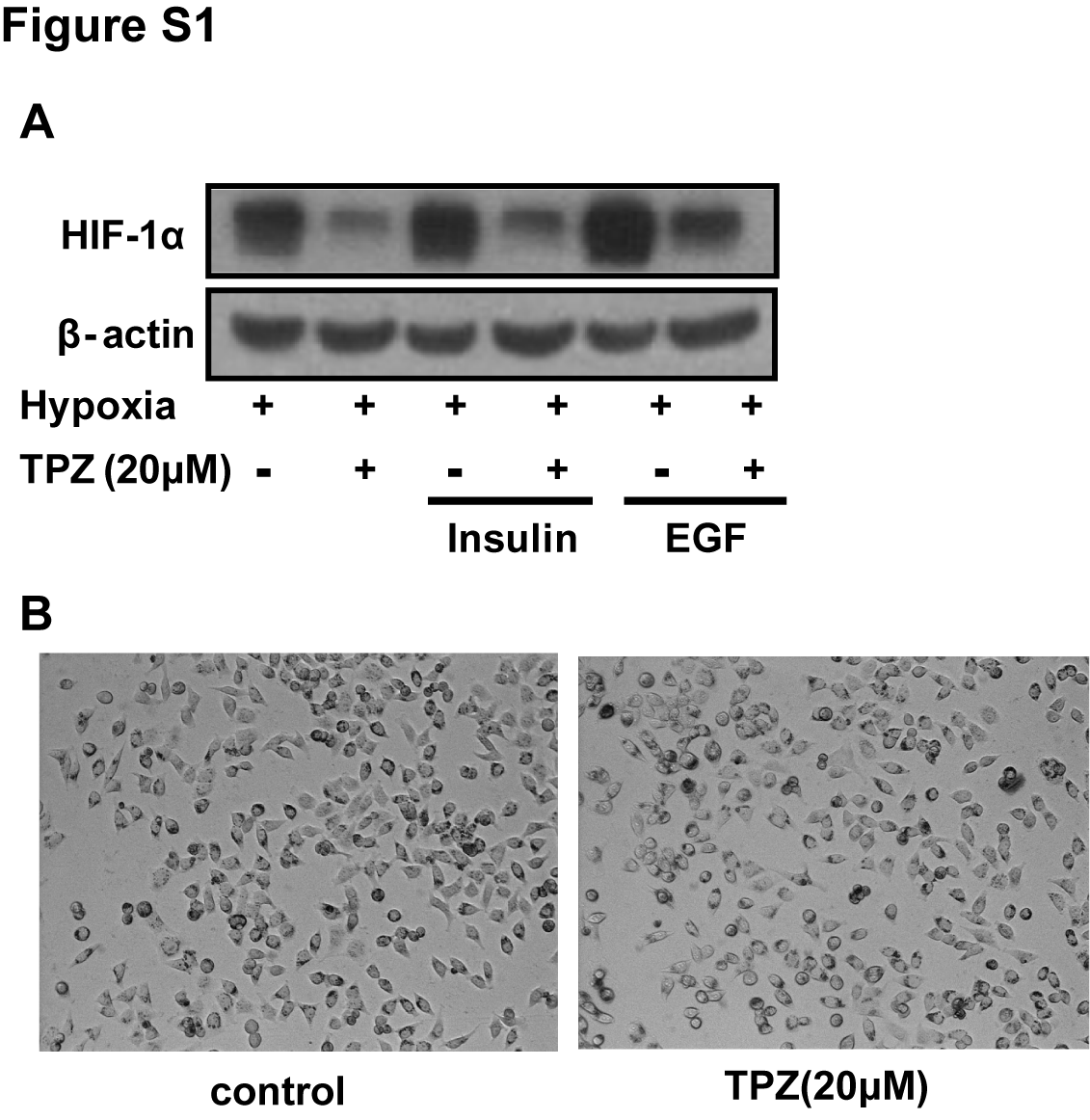

Supplement: Figure S1 — (A) HeLa cells were exposed to 20 µM TPZ after being stimulated by epidermal EGF (100 ng/mL) or insulin (80 U/L) for 4 h at hypoxia. Whole-cell extracts were subjected to immunoblot analysis. (B) Cells were treated with TPZ under hypoxic conditions for 4 h and viewed by microscope. Cell viability was not significantly altered. (0.90 MB TIF) [file pone.0013910.s001.tif]

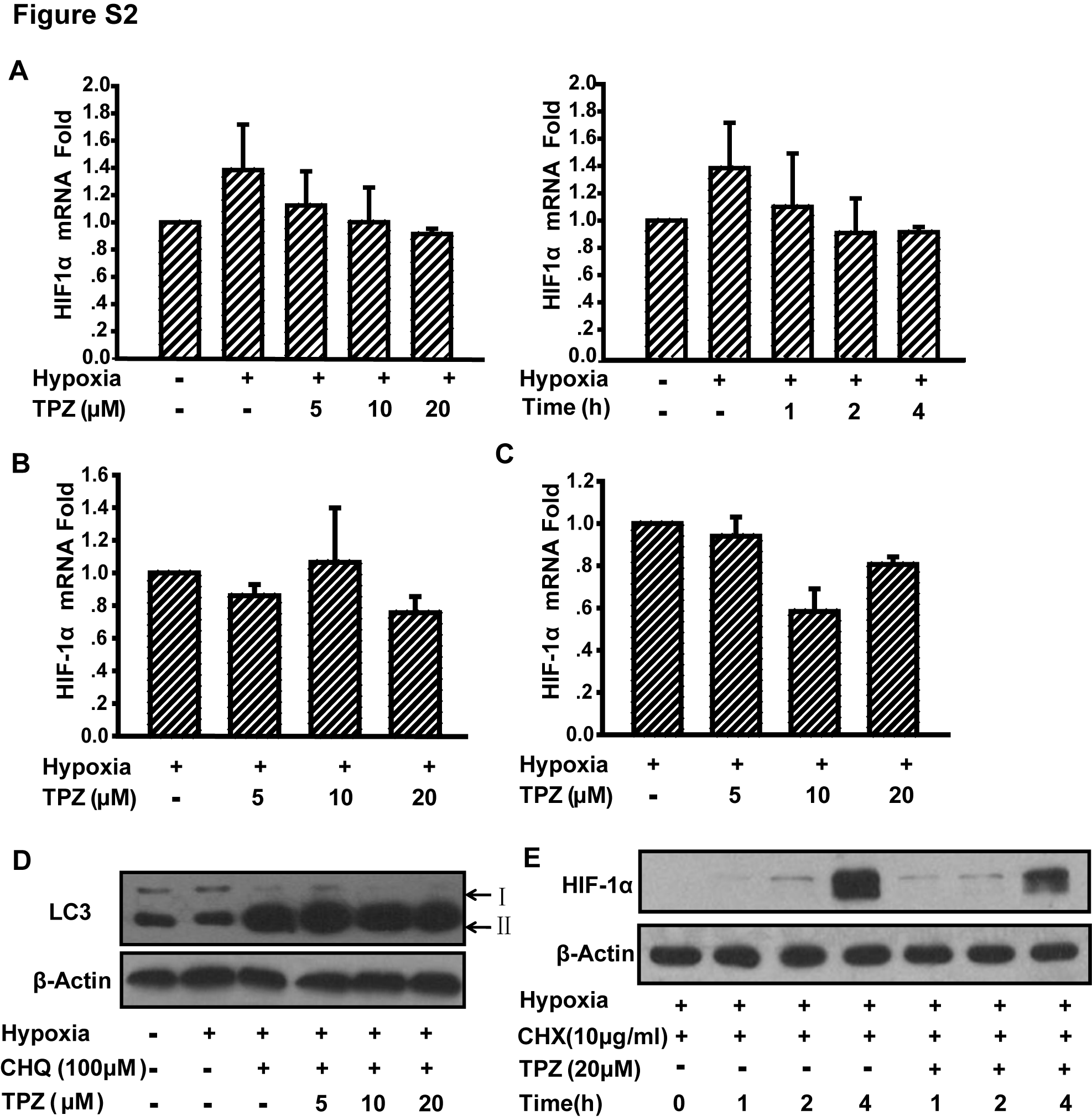

Supplement: Figure S2 — (A) HeLa cells were exposed to varying concentrations of TPZ for 4 h or a single concentration for the indicated times. HIF-1α mRNA levels were determined by real-time PCR. The relative fold change of HIF-1α mRNA compared to GAPDH mRNA in untreated cells under normoxia was arbitrarily set as 1.0. HCT116 cells (B) and A549 cells (C) were exposed to indicated concentrations of TPZ for 4 h at hypoxia. HIF-1α mRNA levels were determined by real-time PCR. The relative fold change of HIF-1α mRNA compared to GAPDH mRNA in untreated cells was arbitrarily set as 1.0. (D) HeLa cells were treated with TPZ, together with chloroquine diphosphate (CQ), under the indicated conditions. The cells were harvested and lysates were immunoprecipitated with an LC3B antibody. The conversion of LC3-I to the lower migrating form LC3-II have been used as a indicator of functional inhibition of the lysosome. (E) HepG2 cells were pre-incubated with CHX for 3 h in normal conditions and then placed in fresh medium and treated with or without 20 µM TPZ for the indicated times under hypoxic conditions. The cells were harvested and lysates were immunoblotted with an HIF-1α antibody. (0.91 MB TIF) [file pone.0013910.s002.tif]

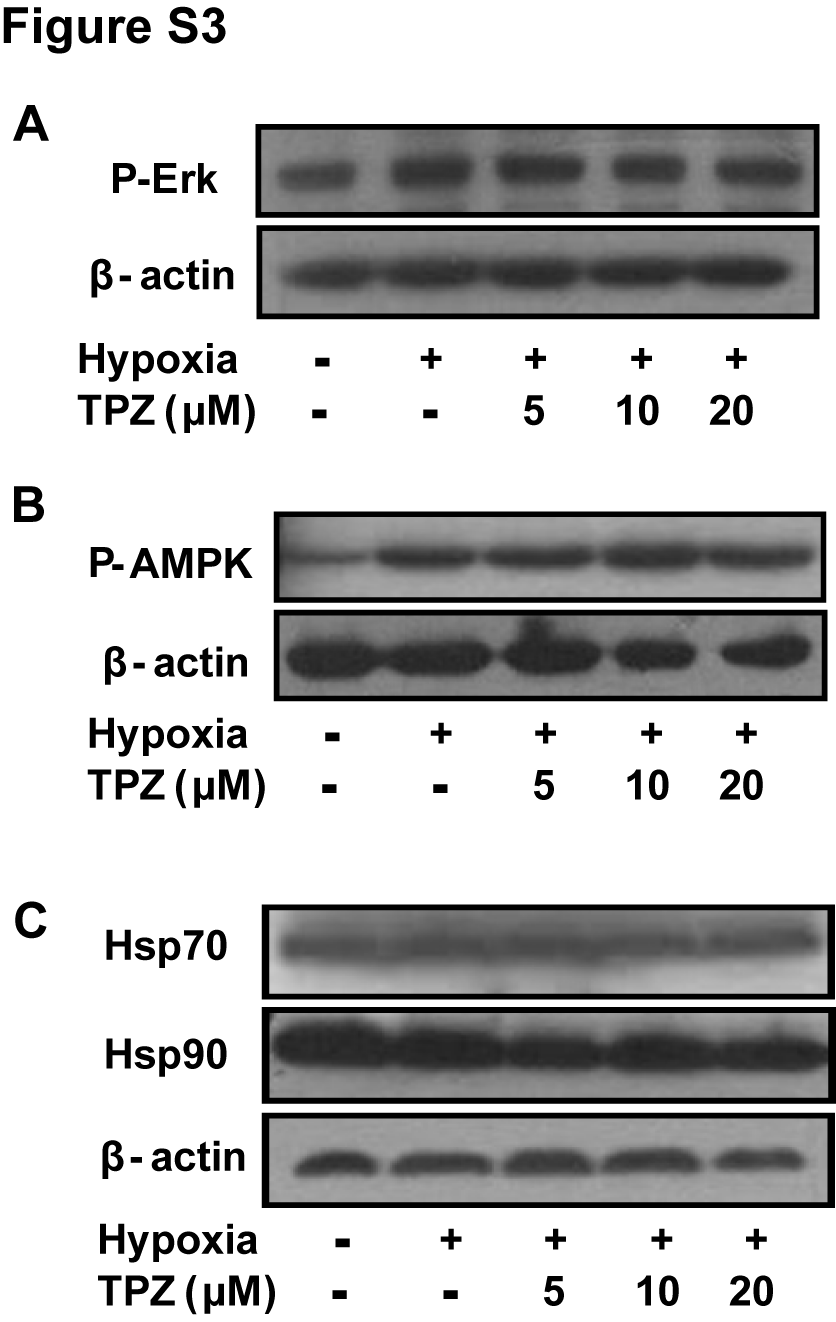

Supplement: Figure S3 — TPZ does not affect the Erk and AMPK pathways and Hsp-family proteins. (A–C) HeLa cells were treated with the indicated concentrations of TPZ at hypoxia for 4 h. Then, the cells were collected and detected for western blotting using specific antibodies. (0.43 MB TIF) [file pone.0013910.s003.tif]

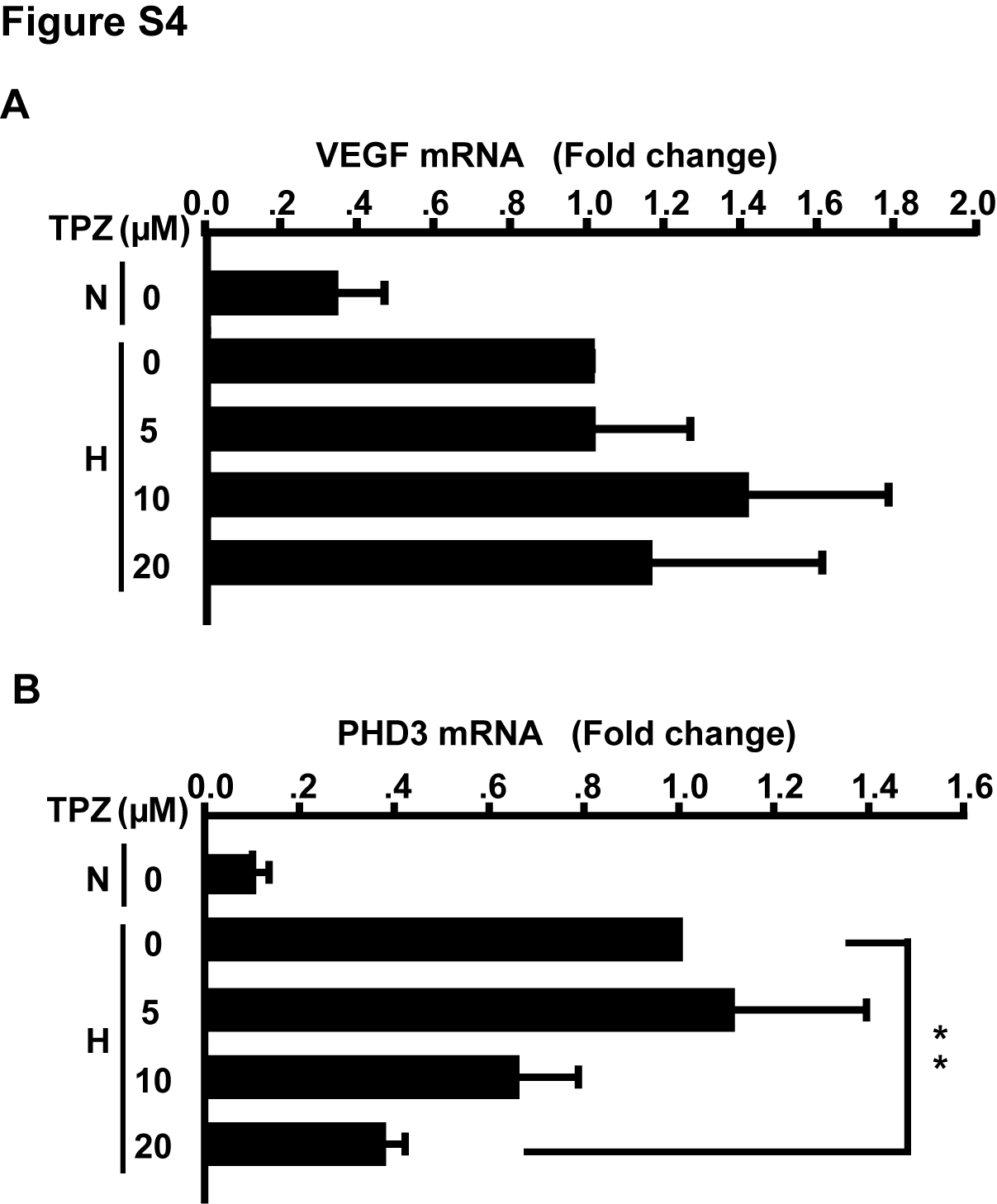

Supplement: Figure S4 — Effects of TPZ on HIF-1α target genes. (A–B) HeLa cells were exposed to varying concentrations of TPZ for 4 h. PHD3 and VEGF mRNA levels were determined by real-time PCR. The relative fold changes of PHD3 and VEGF mRNA compared to GAPDH mRNA in untreated cells under hypoxia was arbitrarily set as 1.0. (0.24 MB TIF) [file pone.0013910.s004.tif]

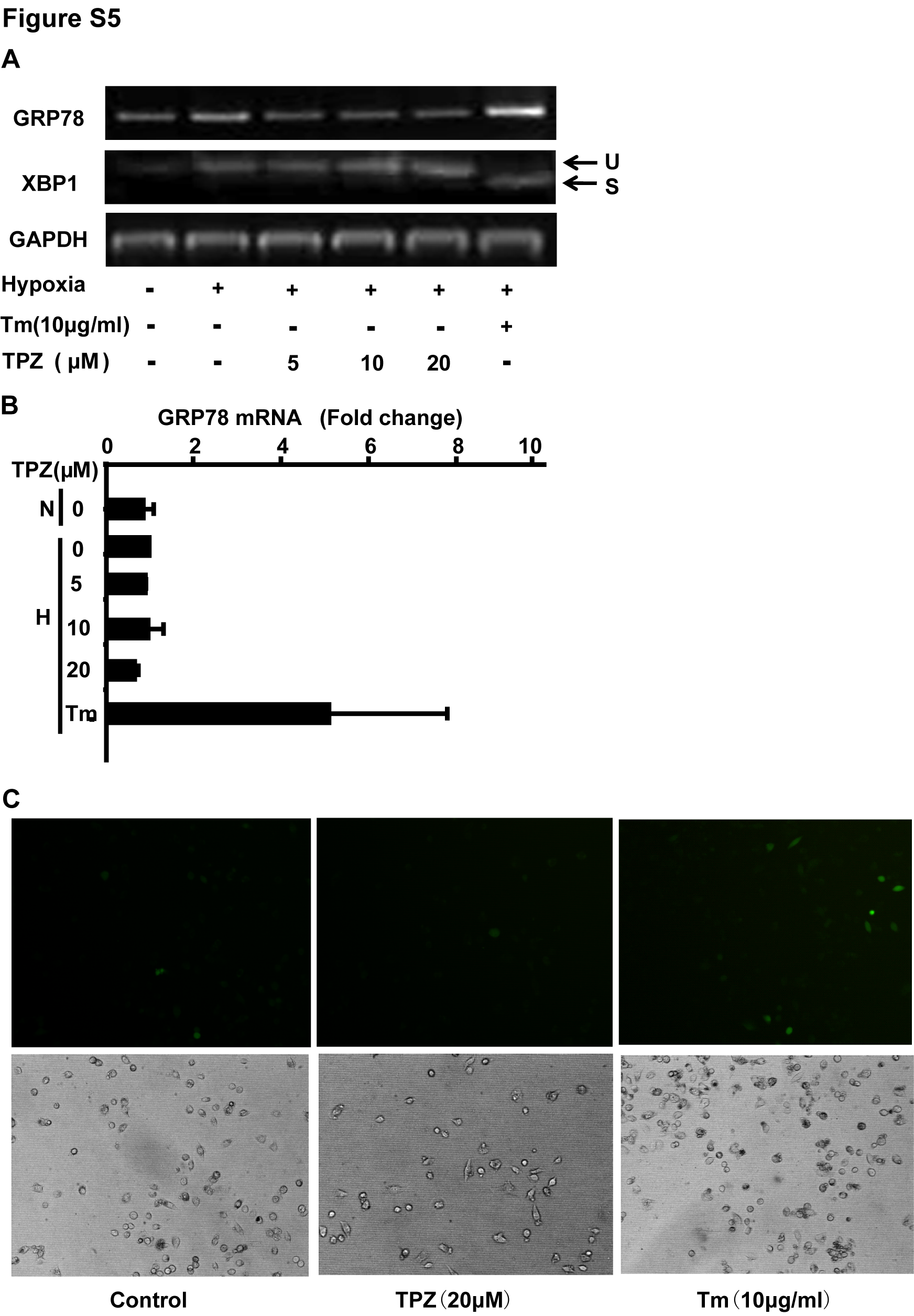

Supplement: Figure S5 — TPZ does not cause ER stress or activate the transcription-dependent branch of the UPR. (A–B) RT-PCR analysis showing induction of UPR targets Grp78 and the appearance of spliced Xbp1 by Tm, but not by TPZ treatment of HeLa cells. (C) HeLa cells were transfected with Xbp1-DBD plasmid and then treated with 20 µM TPZ or 10 µg/mL Tm for 4 h under hypoxic conditions. Fluorescent images were obtained by fluorescence microscope. (2.24 MB TIF) [file pone.0013910.s005.tif]
